# Fas signaling promotes chemoresistance in gastrointestinal cancer by up-regulating P-glycoprotein

**DOI:** 10.18632/oncotarget.2498

**Published:** 2014-10-15

**Authors:** Haoxuan Zheng, Zhizhong Liu, Tao Liu, Yidong Cai, Yadong Wang, Shiyong Lin, Jinmin Chen, Jing Wang, Zhiqing Wang, Bo Jiang

**Affiliations:** ^1^ Guangdong Provincial Key Laboratory of Gastroenterology, Department of Gastroenterology, Nanfang Hospital, Southern Medical University, Guangzhou 510515, China; ^2^ Department of Gastroenterology, the Second People's Hospital of Zhuhai, Zhuhai 519020, China; ^3^ Department of Gastroenterology, Xiyuan Hospital, China Academy of Chinese Medical Sciences, Beijing 100091, China; ^4^ Department of Endoscopy and Laser, Sun Yat-sen University Cancer Center; State Key Laboratory of Oncology in South China; Collaborative Innovation Center for Cancer Medicine, Guangzhou 510060, China

**Keywords:** Fas signaling, epithelial-mesenchymal transition, chemoresistance, gastrointestinal cancer

## Abstract

Fas signaling promotes metastasis of gastrointestinal (GI) cancer cells by inducing epithelial-mesenchymal transition (EMT), and EMT acquisition has been found to cause cancer chemoresistance. Here, we demonstrated that the response to chemotherapy of GI cancer patients with higher expression of FasL was significantly worse than patients with lower expression. Fas-induced activation of the ERK1/2-MAPK pathway decreased the sensitivity of GI cancer cells to chemotherapeutic agents and promoted the expression of P-glycoprotein (P-gp). FasL promoted chemoresistance of GI cancer cell via upregulation of P-gp by increasing β-catenin and decreasing miR-145. β-catenin promoted P-gp gene transcription by binding with P-gp promoter while miR-145 suppressed P-gp expression by interacting with the mRNA 3′UTR of P-gp. Immunostaining and qRT-PCR analysis of human GI cancer samples revealed a positive association among FasL, β-catenin, and P-gp, but a negative correlation between miR-145 and FasL or P-gp. Altogether, our results showed Fas signaling could promote chemoresistance in GI cancer through modulation of P-gp expression by β-catenin and miR-145. Our findings suggest that Fas signaling-based cancer therapies should be administered cautiously, as activation of this pathway may not only lead to apoptosis but also induce chemoresistance.

## INTRODUCTION

Fas (APO-1/CD95) is a member of the TNF and NGF transmembrane receptor superfamily and activates caspase-dependent apoptosis in susceptible cells when triggered by its cognate ligand, FasL/CD95L [1]. However, Fas signaling also controls non-apoptotic cell events including the regulation of cell cycle progression [2], cytokine and chemokine expression [3, 4], tumor growth [5] and motility [6], through various cellular signaling pathways, such as NFκB [6] and MAPK [4, 6]. Moreover, we recently reported that Fas signaling promotes motility and metastasis through inducing the epithelial-mesenchymal transition (EMT) in gastrointestinal (GI) cancer [7].

EMT is a complex molecular and cellular program by which epithelial cells shed their differentiated characteristics, including cell-cell adhesion, planar and apical-basal polarity, and lack of motility, but acquire instead mesenchymal features, including motility, invasiveness and a heightened resistance to apoptosis [8]. EMT has also been found to result in stem cell-like characteristic cells that have a propensity to invade surrounding tissues and display resistance to certain therapeutic interventions [8]. EMT can be triggered by different kinds of stimuli [8], some of which ultimately lead to the upregulation of transcription factors like Twist, Snail, and Slug [9]. Overexpression of these transcriptional factors has been linked to resistance to chemotherapeutics, while depletion of them has been found to increase drug sensitivity [10], suggesting that different stimulus-induced EMT may contribute to chemoresistance via distinct mechanisms. Though Fas signaling was found to induce EMT, it is still unknown whether Fas signaling plays a role in the chemoresistance of cancer cells.

Chemotherapy is the main treatment option for patients with advanced GI cancer. However, cancer cells often become refractory to chemotherapeutic agents owing to the acquirement of multidrug resistance (MDR) [11]. One of the major mechanisms of multidrug resistance is the expression of ATP-binding cassette superfamily proteins (ABC transporters). ABC transporters are ubiquitously and heterogeneously expressed in various body tissues and important pharmacological barriers, which play a pivotal role in host cell detoxification and protection of the body against xenobiotics [11]. ABC transporters can actively efflux a variety of clinical drugs like estramustine, mitoxantrone, and anthracyclines [11]. Overexpression of ABC transporter has been found to correlate with aggressive and invasive cancers, which tend to be more chemoresistant [12]. Sixteen ABC transporters have been identified in MDR [12]. The ABCB1 transporter, also known as P-glycoprotein (P-gp), is encoded by human multidrug resistance 1 gene (*MDR1*) and is an important mediator of drug resistance [13]. P-gp has been found to be correlated with the intrinsic and acquired drug resistance in several neoplasms and represents the failure of chemotherapeutic treatments and poor prognosis of cancer [13]. Whether Fas signaling promotes cancer chemoresistance via MDR in gastrointestinal (GI) cancer remains to be determined.

In this study, we have investigated the association of Fas signaling with chemoresistance in GI cancer and explored the underlying mechanisms both *in vitro* and *in vivo*. We found that Fas activation promoted the chemoresistance in GI cancer through induction of P-gp expression via upregulating nuclear β-catenin and downregulating miR-145 expression.

## RESULTS

### Activation of Fas signaling may result in chemoresistance of GI cancer

**Figure 1:**
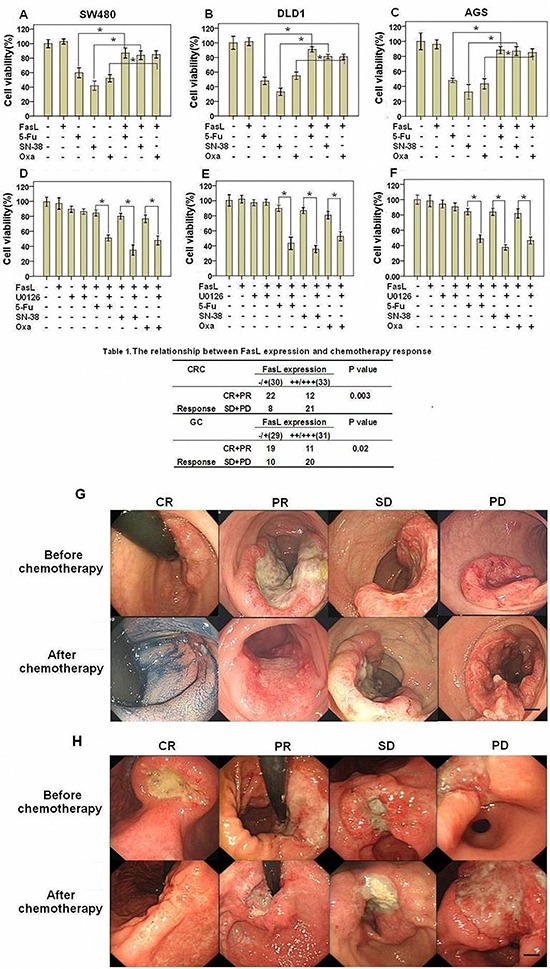
Fas activation confers GI cancer cells with chemoresistance SW480 **(A, D)**, DLD1 **(B, E)**, and AGS **(C, F)** cells were seeded in 96-well plate (10000 cells/well), cultured overnight and then treated with FasL for 24 hours. After that, cells were treated with different drug as indicated for additional 48 hours, and cell viability was analyzed. U0126 was added 2 hours before FasL stimulation. Size changes in primary CRC **(G)** and GC **(H)** examined by endoscopy were also shown. **(A-F)** Data are represented as fold-change ± SD compared to control cells. *In vitro* experiments were performed in triplicate. *P < 0.05. CRC: colorectal cancer; GC: gastric cancer; CR: complete response; PR: partial response; SD: stable disease; PD: progressive disease. Scale bar: 5 mm.

Recently, we reported that Fas signaling promoted the motility and metastasis of GI cancer cells through induction of EMT [7]. Besides the increased motility and metastasis, cancer cells acquiring EMT may have an enhanced resistance to chemotherapeutics-induced apoptosis [8]. To test this hypothesis, we stimulated GI cancer SW480, DLD1 and AGS cells with FasL before treatment of chemotherapeutic agents (5-Fu, SN38, or Oxaliplatin) and then detected cell viability. FasL alone did not apparently affect cell viability. Either 5-Fu, SN38, or Oxaliplatin significantly reduced the cell viability in all the three cell lines. Interestingly, we found that the cell viability was significantly higher in GI cancer cells treated with FasL in advance than that treated with drugs directly (Figures 1A, 1B and 1C), indicating that activation of Fas signaling confers GI cancer cells resistant to chemotherapies. The ERK1/2 MAPK pathway is activated by Fas signaling and required for FasL-induced EMT and motility [7]. We asked whether FasL-induced chemoresistance depends on the ERK1/2 MAPK pathway. For this, we additionally treated these cells with a MEK1/2 specific inhibitor U0126 or PD98095 (data not shown) and found that U0126 or PD98095 (data not shown) significantly abolished the FasL-induced resistance to 5-Fu, SN38, or Oxaliplatin (Figures 1D, 1E and 1F). These data showed that activation of Fas signaling confers GI cancer cells resistant to chemotherapies in an ERK1/2 MAPK pathway dependent way.

**Table 1:** The relationship between FasL expression and chemotherapy response

CRC		FasL expression		P value
		−/+(30)	++/+++(33)	
Response	CR+PR	22	12	0.003
	SD+PD	−8	21	
**GC**		**FasL expression**		**P value**
		**−/+(29)**	**++/+++(31)**	
Response	CR+PR	19	11	0.02
	SD+PD	10	20	

To extend our above observation in clinic, we investigated the relationship between chemotherapy (FOLFIRI: 5-FU, leucovorin, irinotecan) response and the expression status of FasL in GI cancer specimens examined by both qRT-PCR and immunohistochemitry. In both CRC and GC, patients with lower expression of FasL responded better to chemotherapy than patients with higher expression of FasL (Table 1, Figure 1G and 1H), indicating that Fas signaling may contribute to chemoresistance of GI cancer.

### P-glycoprotein is a key transporter for FasL-induced chemoresistance

**Figure 2:**
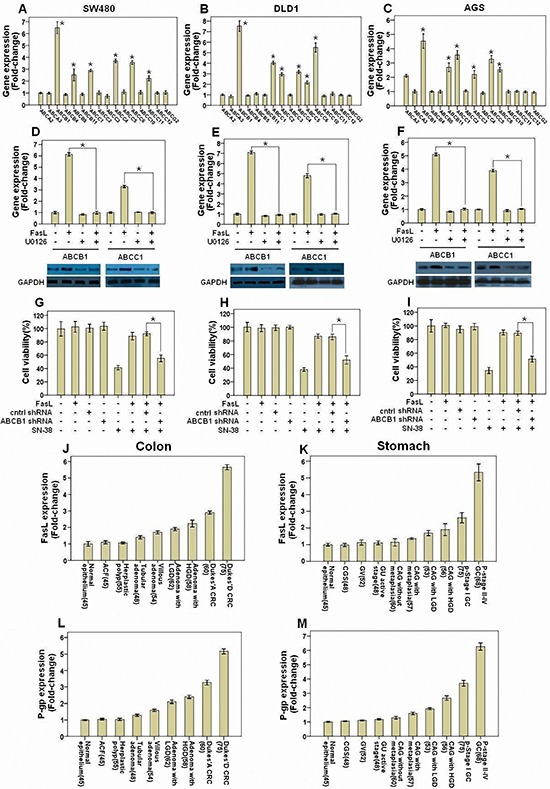
Fas signaling promotes chemoresistance by inducing P-gp expression in GI cancer cells SW480 **(A, D)**, DLD1 **(B, E)**, and AGS **(C, F)** were treated with or without FasL for 24 or 72 hours. qRT-PCR or immunoblot were performed to detect the mRNA and proteins of the indicated genes. U0126 was added 2 hours before FasL stimulation. SW480 **(G)**, DLD1 **(H)**, and AGS **(I)** cells stably expressing ABCB1 or control shRNA construct were seeded in 96-well plate (10000 cells/well) and cultured overnight, and then treated with FasL for 24 hours. After that, cells were treated with SN-38 for 48 hours, and then cell viability was analyzed. Human GI precancerous (J, colon and rectum, N=367; K, Stomach, N=419) and cancer samples (L, CRC, N=135; M, GC, N=143) were analyzed by qRT-PCR. Sample number is indicated in parentheses. All data are represented as fold-change ± SD compared to control cells or group. Experiments were performed in triplicate for *in vitro* studies. *P < 0.05.

ABC transporters actively efflux a wide spectrum of commonly employed chemotherapeutic drugs. Overexpression of ABC transporters plays a key role in the chemoresistance in lots of cancers [11, 12]. We proposed that Fas activation might induce expression of certain ABC tranporters to acquire chemoresistance. With qRT-PCR-based analysis, we assessed the mRNA levels of the 16 ABC transporters, which were found to be implicated in drug resistance [12], in SW480, DLD1 and AGS cells after FasL stimulation. We found that the 16 ABC transporters were heterogeneously expressed in GI cells with ABCB1, ABCC1 and ABCC6 overexpressed in SW480 (Figure 2A), DLD1 (Figure 2B) and AGS cells (Figure 2C) following Fas signaling activation. Among them, ABCB1 (P-gp) expression was significantly upregulated much more than the other ABC transporters (Figures 2A, 2B and 2C). Consistent with the induction of mRNA levels, immunoblots showed apparent upregulation of the protein levels of ABCB1 and ABCC1 by FasL. Further, inhibition of ERK1/2 MAPK pathway by U0126 abolished the induction of both ABCB1 and ABCC1 by FasL in SW480 (Figure 2D), DLD1 (Figure 2E) and AGS cells (Figure 2F). It has been shown that P-gp is responsible for the resistance of cells to chemotherapy drug SN-38, an active metabolite of irinotecan. We next knocked down P-gp by lentiviral shRNA and treated with SN-38 in GI cancer cells. P-gp shRNA significantly reduced FasL-induced chemoresistance in SW480 (Figure 2G), DLD1 (Figure 2H) and AGS cells (Figure 2I). Moreover, compared to control lentiviral shRNA, *ABCB1* lentiviral shRNA significantly increased the accumulation of rhodamine 123, a substrate of P-gp, following treatment with FasL in GI cancer cells (Supplementary information, Figures S1A, S1B and S1C, S1D, S1E and S1F). Besides *ABCB1* lentiviral shRNA, Tariquidar, a highly selective and potent non-competitive inhibitor of P-gp, was also utilized in SN-38 cell viability assay and rhodamine 123 efflux assay. Similar results as use of *ABCB1* lentiviral shRNA were obtained in both assays (data not shown). Taken together, these results indicate that P-gp is a key factor for FasL-induced chemoresistance.

**Figure 3:**
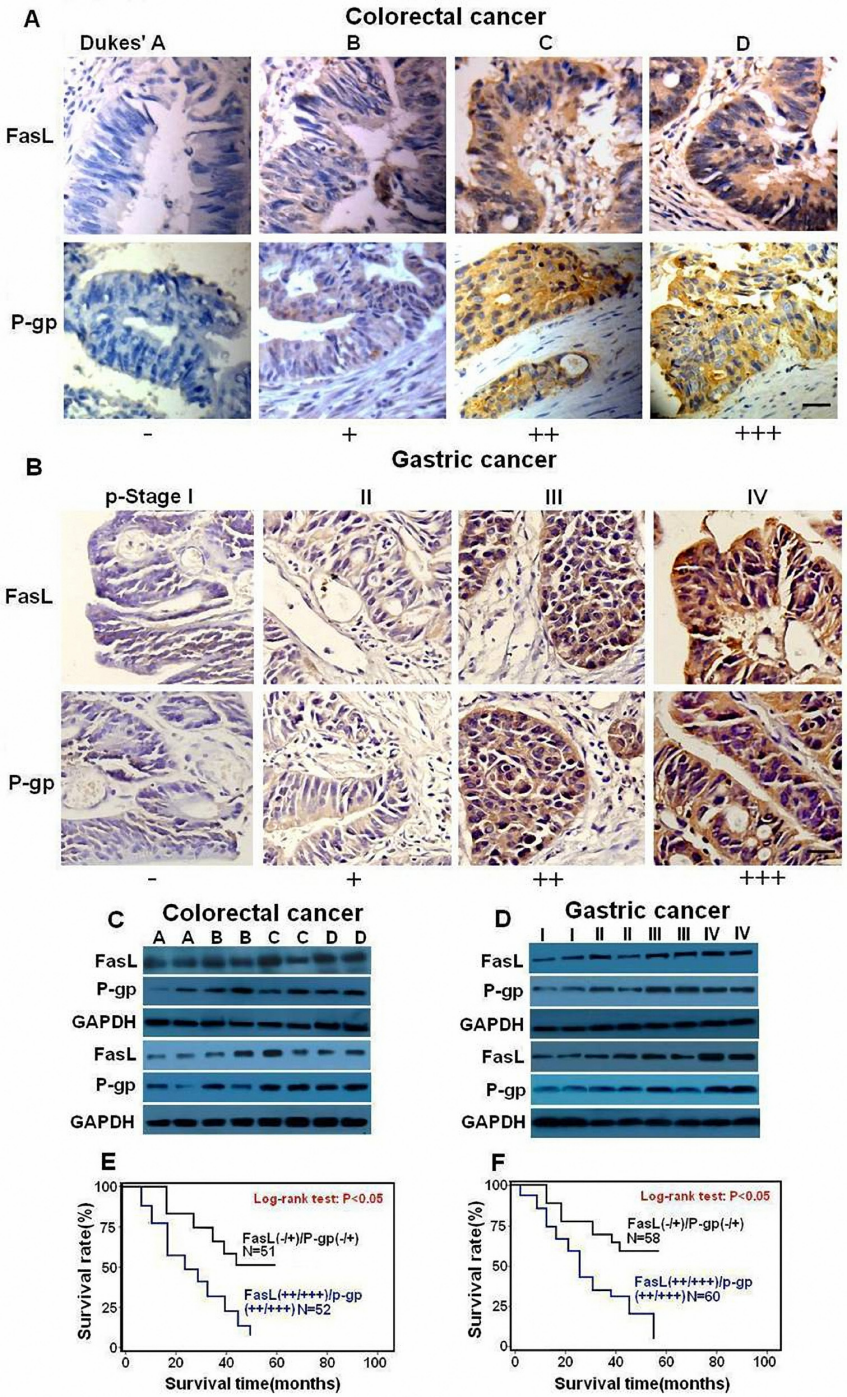
FasL and P-gp are overexpressed in human GI cancer cells Immunostaining (×400) and immunoblot for FasL and P-gp were conducted with human CRC **(A, C)** and GC **(B, D)** samples. 2 representative samples from each stage **(C, D)** are tested by immunoblot. The expression of both FasL and P-gp increased during GI cancer progression. Survival curves were generated according to follow-up data with Kaplan-Meier method, and comparison between cumulative survival rates was performed using log-rank test. CRC (E) and GC (F) patients with higher expression (++ /+++) of both molecules showed worse prognosis than patients with lower expression (−/+). Scale bar: 50 μm.

We next explored the relationship between Fas signaling and P-gp expression level in GI cancer tissues. We detected the mRNA and protein levels of FasL and P-gp by qRT-PCR, immunoblot and immunohistochemistry in fresh GI precancerous and cancer samples. The mRNA levels of FasL and P-gp showed a gradual upregulation in precancerous samples but a sharp increase in various stages of CRC (Figures 2J and 2L) and GC (Figures 2K and 2M). In consistence, immunohistochemistry analysis showed a gradual increase of of both FasL and P-gp proteins in CRC (Figure 3A) and GC (Figure 3B), with the highest expression of FasL and P-gp in the advanced stage D in CRC and stage IV in GC. Similar patterns of the alteration of the protein levels of both FasL and P-gp in CRC (Figure 3C) and GC (Figure 3D) were also revealed by immunoblots (Supplementary information, Figures S2A, S2B, S2C, S2D, Tables S5 and S6). Most importantly, a positive correlation between FasL and P-gp was noted in both mRNA and protein levels (Supplementary information, Tables S3, S4, S7 and S8). If combining FasL and P-gp according to rank by the immunohistochemistry, patients with higher expression (++/+++) of both molecules showed worse prognosis than patients with lower expression (−/+) in CRC (Figure 3E) and GC (Figure 3F). Altogether, these data imply that Fas signaling may promote P-gp expression in GI cancer *in vivo*.

### β-catenin promotes P-gp expression by binding with P-gp promoter

**Figure 4:**
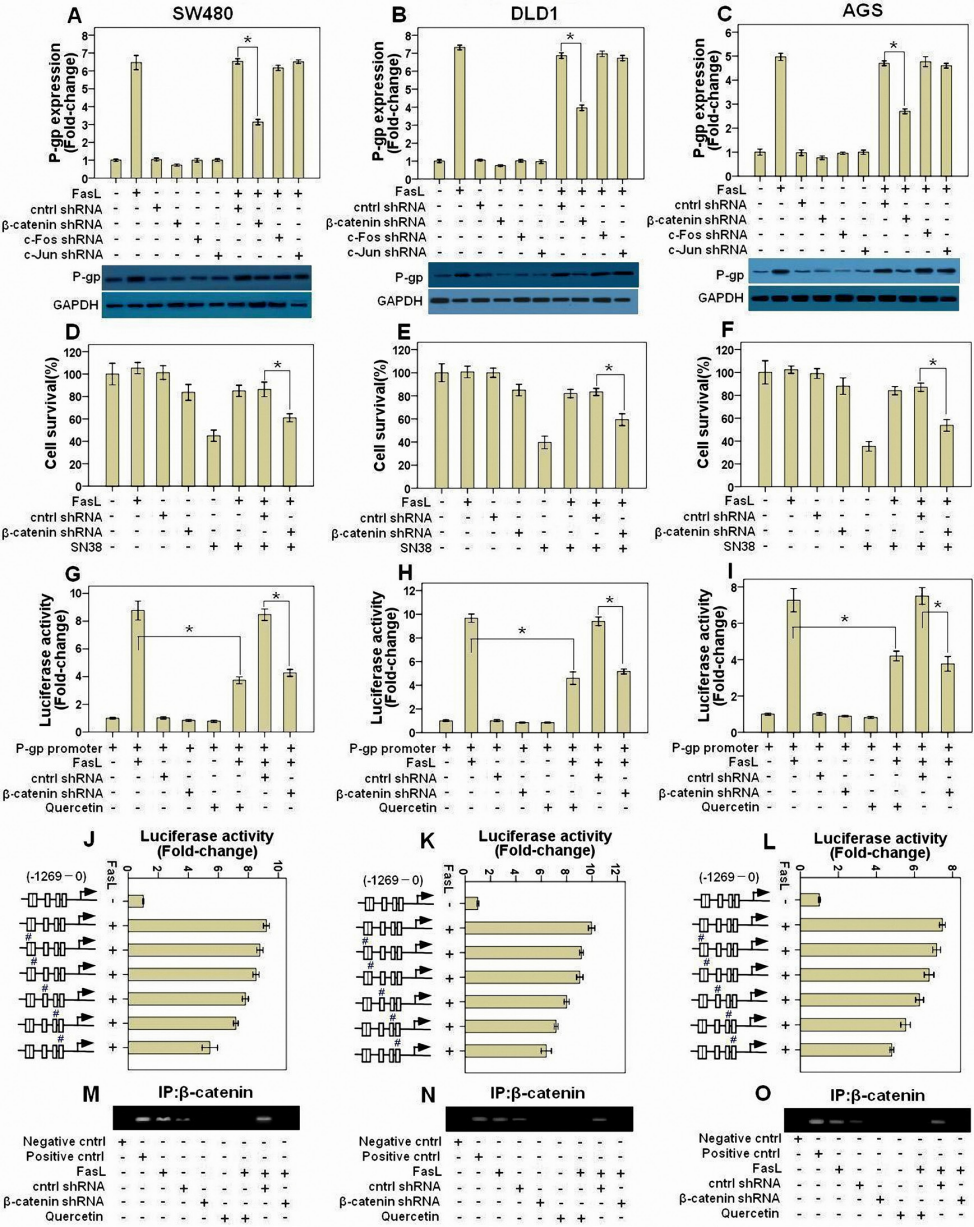
FasL-induced β-catenin activity promotes P-gp expression by binding with its promoter SW480 **(A)**, DLD1 **(B)**, and AGS **(C)** cells stably expressing *β-catenin*, *c-Jun*, *c-Fos*, or control shRNA construct were treated with FasL for 24 or 72 hours, and then qRT-PCR or immunoblot was conducted to detect the mRNA and proteins of the indicated genes. SW480 **(D)**, DLD1 **(E)**, and AGS **(F)** cells stably expressing *β-catenin* or control shRNA construct were seeded in 96-well plate (10000 cells/well) and cultured overnight, and then treated with FasL for 24 hours. After that, cells were treated with SN-38 for 48 hours, and then cell viability was analyzed. P-gp promoter reporter was transduced into SW480 **(G)**, DLD1 **(H)**, and AGS **(I)** cells stably expressing either *β-catenin* or control shRNA construct, and luciferase activity was assessed after FasL treatment for 12 hours. The 5 predicted binding sites for β-catenin on P-gp promoter were mutated one by one using Quick Change Site-Directed Mutagenesis Kit, as indicated with “**#**” in J, K, L. Luciferase activity was assayed after FasL treatment for 12 hours. SW480 **(M)**, DLD1 **(N)**, and AGS **(O)** cells stably expressing *β-catenin* or control shRNA construct were stimulated with FasL for 1 hours and ChIP assay was performed with β-catenin antibody. E-cadherin antibody was used as negative control, while products amplified from the P-gp promoter were used as positive control. Quercetin (10 μM) was added 2 hours before FasL stimulation. **(A-L)** Data are represented as fold-change ± SD compared to control cells. Experiments were performed in triplicate. *P < 0.05.

FasL-induced EMT is accompanied with the activation of AP-1 complex and β-catenin [14, 15]. Moreover, there are several binding sites for both AP-1 complex and β-catenin in P-gp gene promoter[16, 17]. These facts led us to hypothesize that FasL may promote the gene expression of P-gp via AP-1 complex and/or β-catenin. To test this proposal, we downregulated *c-Jun*, *c-Fos* and *β-catenin* by shRNA in GI cancer cells and checked P-gp levels in the presence or absence of FasL. We found that knockdown of β-catenin but not AP-1 significantly inhibited FasL-induced upregulation of both the mRNA and protein levels of P-gp in SW480 (Figure 4A), DLD1 (Figure 4B) and AGS cells (Figure 4C). In support of these results, *β-catenin* shRNA significantly abolished FasL-induced resistance to SN-38 in SW480 (Figure 4D), DLD1 (Figure 4E) and AGS cells (Figure 4F). Moreover, in the luciferase reporter assays, FasL significantly induced the activity of P-gp promoter, which was apparently inhibited by *β-catenin* but not control shRNA constructs. In addition, inhibition of the transcriptional activity of β-catenin/TCF by quercetin also significantly abolished the induction of the activity of P-gp promoter by FasL in SW480 (Figure 4G), DLD1 (Figure 4H) and AGS cells (Figure 4I). These results clearly demonstrated that β-catenin is responsible for FasL-mediated upregulation of the P-gp. Following mutating the 5 predicted binding sites for β-catenin on P-gp promoter, we found that the first one next to the transcription starting site (TTS) played the most important role in the induction of P-gp expression by FasL (Figures 4J, 4K and 4L). Further ChIP assay demonstrated that FasL promoted the binding of β-catenin to the first binding site next to TTS in P-gp promoter, which was abolished by either quercetin or *β-catenin* but not control shRNA (Figures 4M, 4N and 4O).

### The Fas- ERK1/2-GSK3β- β-catenin signaling axis controls the gene expression of P-gp

β-catenin is negatively controlled by GSK3β, which can be inhibited by ERK1/2 MAPK. In our previous study [14], we showed that the nuclear expression and transcriptional activity of β-catenin were increased by inhibitory phosphorylation of GSK-3β at Ser 9 by FasL-induced ERK1/2 MAPK signaling. These facts suggest that FasL promote P-gp expression via the Fas- ERK1/2-GSK3β- β-catenin signaling axis. Indeed, we found that exogenous expression of GSK-3β S9A mutant, which can't be inactivated by phosphorylation at Ser9, partially prevented the FasL-induced changes of P-gp expression (Supplementary information, Figures S3A, S3B and S3C), chemoresistance (Supplementary information, Figures S3D, S3E and S3F) and P-gp promoter activity (Supplementary information, Figures S3G, S3H and S3I) in GI cancer cells. These results indicate P-gp expression is also controlled by GSK-3β that is inhibited by FasL-induced ERK1/2 MAPK signaling.

**Figure 5:**
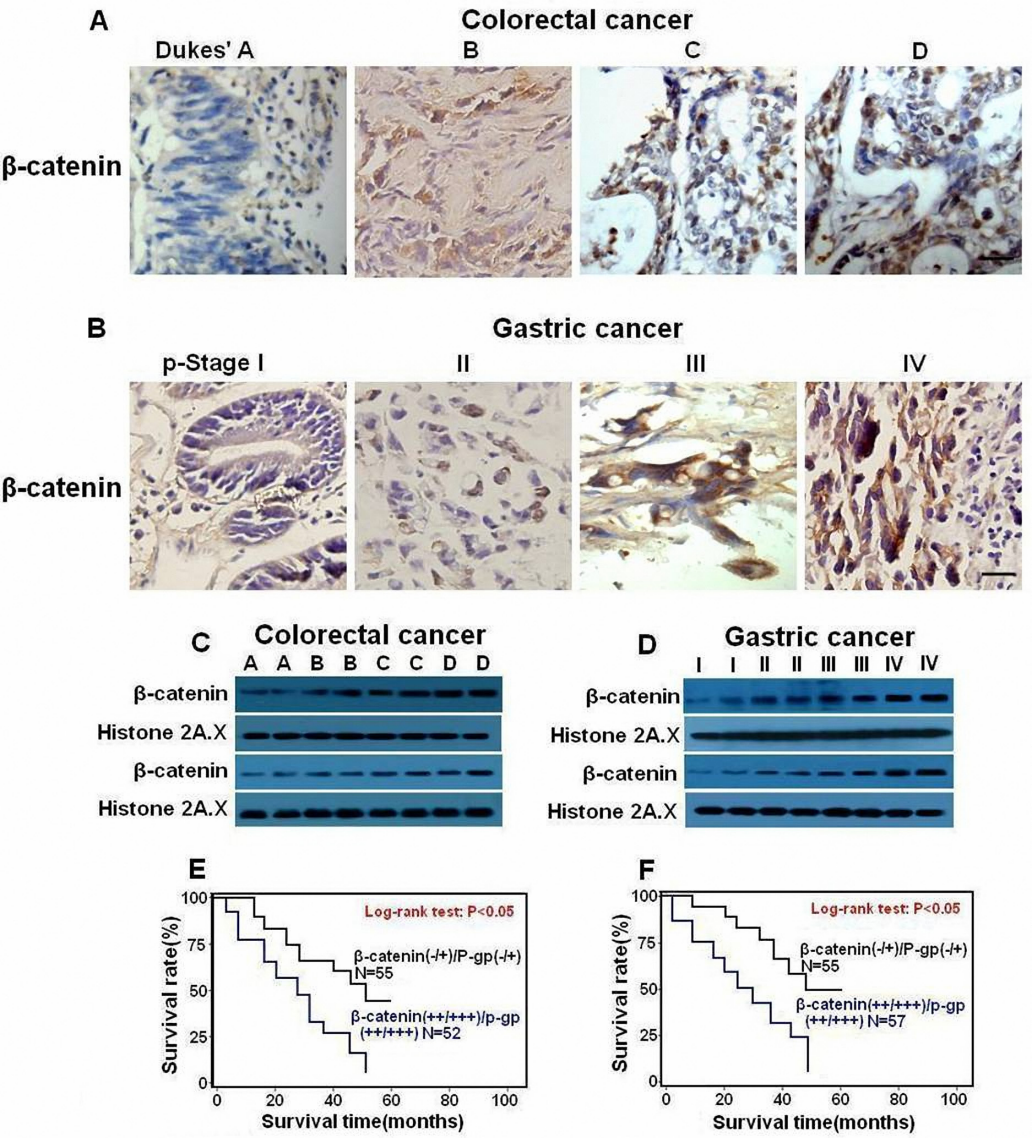
Nuclear expression of β-catenin is upregulated in human GI cancer cells and correlated with shorter survival of GI patients Immunostaining (×400) and immunoblot for β-catenin was conducted with human CRC **(A,C)** and GC **(B,D)** samples. Expression of β-catenin increased during GI cancer progression. Survival curves were generated according to follow-up data with Kaplan-Meier method, and comparison between cumulative survival rates was performed using log-rank test. CRC **(E)** and GC **(F)** patients with higher expression (++/+++) of β-catenin and P-gp showed worse prognosis than patients with lower expression (−/+). Scale bar: 50 μm.

In order to investigate whether Fas signaling promotes P-gp expression by upregulating nuclear β-catenin expression *in vivo*, fresh GI cancer samples were analyzed by immunohistochemistry and immunoblot. With the progression of tumorigenesis, enhanced nuclear staining of β-catenin was observed in both CRC (Figure 5A) and GC (Figure 5B, Supplementary information, Tables S5 and S6). Similarly, apparently increased protein levels of β-catenin were detected in the later stages of CRC (Figures 5C, Supplementary information, Figure S2E) and GC (Figures 5D, Supplementary information, Figure S2F). Most importantly, a positive correlation between β-catenin and FasL or P-gp was observed according to the ranking by immunohistochemistry (Supplementary information, Tables S7 and S8). Moreover, when β-catenin and P-gp were combined in accordance with the ranking by the data from immunohistochemistry, patients with higher expression (++/+++) of both β-catenin and P-gp showed worse prognosis than patients with lower expression (−/+) in CRC (Figure 5E) and GC (Figure 5F). Thus, Fas activation may promote P-gp expression at least partially through nuclear β-catenin activity in GI cancer.

### miR-145 suppresses P-gp expression through interaction with the 3′UTR of P-gp mRNA

**Figure 6:**
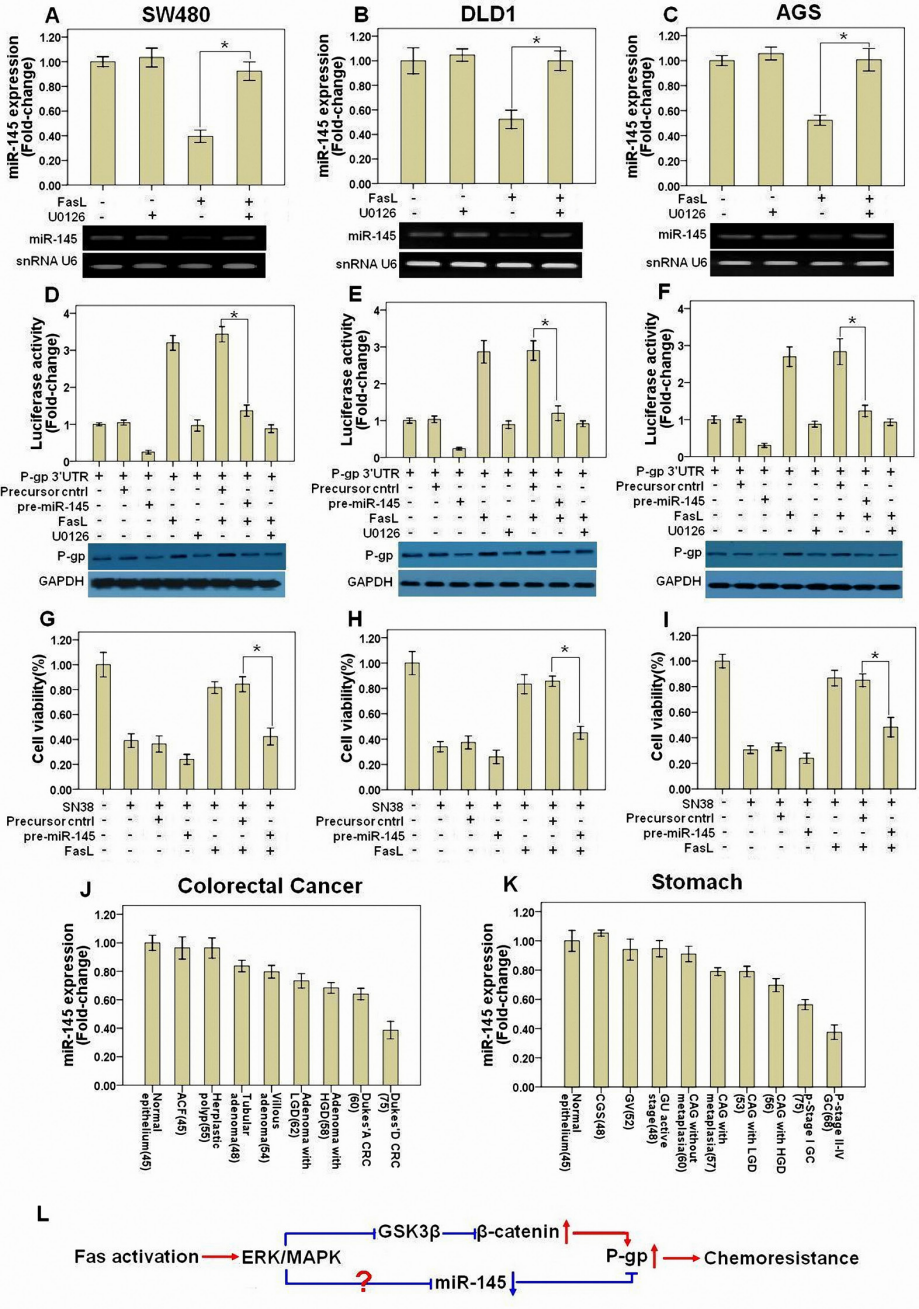
miR-145 is downregulated after FasL-induced ERK1/2 activation and inhibits P-gp expression SW480 **(A)**, DLD1 **(B)**, and AGS **(C)** cells were treated with FasL (12 h) in the presence or absence of U0126. miR-145 levels were determined by qRT-PCR with U6 RNA as internal control. A P-gp 3′UTR reporter was transduced into SW480 **(D)**, DLD1 **(E)**, and AGS **(F)** cells stably expressing either miR-145 precursor or control precursor, and luciferase activity was assessed after treatment with FasL (12 h) and/or U0126. Immunoblot was performed in parallel with FasL stimulation for 72 hours, but without transfection of P-gp 3′UTR reporter. SW480 **(G)**, DLD1 **(H)**, and AGS **(I)** stably expressing miR145 precursor or control precursor were seeded in 96-well plate (10000 cells/well) and cultured overnight, and then treated with FasL for 24 hours. After that, cells were treated with SN-38 for 48 hours, and then cell viability was analyzed. Human GI precancerous (J, colon and rectum, N=367; K, Stomach, N=419) and cancer samples (J, CRC, N=135; K, GC, N=143) were analyzed by qRT-PCR. Sample number is indicated in parentheses. **(L)** The schematic model for how Fas signaling promotes chemoresistance by regulation of P-gp expression was shown. All data are represented as fold-change ± SD compared to control cells or group. Experiments were performed in triplicate for *in vitro* studies. *P < 0.05.

Our above results showed that silencing β-catenin by shRNA or inhibition of β-catenin transcriptional activity by quercetin significantly but not completely abolished the induction of P-gp by FasL, suggesting that there is alternative mechanism by which Fas signaling promotes the expression of P-gp. Recently, several miRNAs including miR-508-5p [18], miR-451 [19], miR-145 [20], miR-298 [21], miR-1253 [21], and miR-338 [21], have been proposed to target the P-gp mRNA 3′UTR and inhibit its translation. We wonder if FasL also increase the level of P-gp by altering the expression of miRNAs that target P-gp. For this, we detected the expression of these miRNAs after FasL treatment in GI cancer cells by qRT-PCR. Among these miRNAs, we found that only miR-145 was downregulated and dependent on FasL-induced ERK1/2 MAPK activation (Figures 6A, 6B and 6C). Ectopic overexpression of miR-145 inhibited P-gp 3′UTR luciferase activity and prevented FasL-induced increase of P-gp 3′UTR reporter activity. Furthermore, overexpression of miR-145 reduced the protein expression of P-gp as well as inhibited FasL-induced upregulation of P-gp (Figures 6D, 6E and 6F), whereas downregulation of miR-145 promoted P-gp expression and repressed cell apoptosis induced by SN-38 (Supplementary information, Figure S4). Additionally, FasL-induced chemoresistance to SN-38 was also inhibited by miR-145 overexpression (Figures 6G, 6H and 6I). Finally, when the miR-145 binding site (seed sequence) in the 3′UTR region of P-gp was mutated, miR-145 precursor or FasL stimulation couldn't decrease or increase the P-gp 3′UTR reporter activity (Supplementary information, Figure S5). These results demonstrated that P-gp is a direct target of miR-145.

To elucidate the functional linkage between miR-145 and P-gp, we detected the expression of miR-145 in fresh GI precancerous and cancer samples by qRT-PCR. The expression of miR-145 decreased smoothly among precancerous samples but sharply in cancer in various stages of CRC (Figure 6J) and GC (Figure 6K; Supplementary information, Tables S1 and S2). Moreover, miR-145 was negatively correlated with FasL or P-gp according to the ranking by qRT-PCR (Supplementary information, Tables S3 and S4). Taken together, these data revealed that Fas signaling induces P-gp expression partially through downregulation of miR-145 expression in GI cancer both *in vivo* and *in vitro*.

## DISCUSSION

EMT process has been found to intricately associate with increased drug resistance; yet, little is known about the molecular mechanisms linking these two phenomena. In this study, with comprehensive clinic analysis and *in vitro* cell culture experiments, we found that the Fas signaling promotes chemoresistance of GI cancer via upregulating P-gp by two distinct mechanisms. Fas signaing enhances the gene transcription of P-gp by increasing β-catenin through ERK1/2 MAPK-GSK3β signaling axis. In addition, this pathway upregulates P-gp expression via decreasing miR-145 (Figure 6L).

Cells undergoing EMT become more mobile and invasive [22], while invasive cancer cells are found to be more resistant to anticancer drugs [23, 24]. Fas signaling has been found to promotes the motility and metastasis of GI cancer through EMT [7]. Our data demonstrate that Fas activation leads to chemoresistance of GI cancer cells and depends on the ERK1/2 MAPK pathway. Accordingly, GI cancer patients with lower expression of FasL responded better to FOLFIRI chemotherapy than patients with higher expression of FasL, although these results still need more samples to verify.

Failure of cancer chemotherapy can occur through increased efflux of chemotherapeutic agents, leading to the reduction of intracellular drug levels and consequent drug insensitivity [11, 12]. A well-established cause of multidrug resistance (MDR) involves the increased expression of the members of ABC transporter superfamily, many of which efflux various chemotherapeutic compounds from cells [11, 12]. The most extensively characterized MDR transporters include ABCB1 (also known as MDR1 or P-gp), ABCC1 (also known as MRP1) and ABCG2 (also known as BCRP or MXR) [12]. P-gp expression reflects the tumour phenotype in colorectal carcinomas in which P-gp levels correlate with invasion into vessels [25]. P-gp is preferentially expressed in poorly differentiated colon tumours, but undetectable in normal colon tissue [26]. Additionally, P-gp is overexpressed in gastric cancer and linked to poor prognosis and MDR [27, 28]. In this study, we found that Fas signaling could promote P-gp expression *in vitro*, and a significant positive correlation between FasL and P-gp was observed in GI cancer samples. However, P-gp was found to correlate with GI cancer stages but not with differentiation. These conflicting results may be due to sample size, patient sources, and different detection methods. Therefore, large cohort of samples from multiple medical centers with uniform analysis methods is needed to draw a confirmative conclusion. Additionally, Fas signaling also promotes expression of other transporters, such as ABCC6, which are probably involved in FasL-induced chemoresistance.

Induction of EMT leads to increased expression of ABC transporters and drug resistance, whereas a reversal of EMT in invasive cells results in a concomitant decrease in ABC transporter expression and chemoresistance, thus establishing a strong linkage between EMT and ABC transporter expression [29]. Moreover, several ABC transporters contain binding sites for EMT regulators like Twist, Snail, Slug, FOXC2, and E12/E47, and at least three of these factors, Snail, Twist, and FOXC2, can modulate the promoter activity of ABC transporters [29]. Previously, we reported that the EMT regulators AP-1 complex and β-catenin were activated during FasL-induced EMT. Coincidently, there are several binding sites for both AP-1 complex and β-catenin in P-gp promoter [16,17]. FasL-induced ERK1/2 MAPK signaling leads to inhibition of GSK3β kinase, which in turn phosphorylates and promotes the degradation of β-catenin via ubiquitin-proteasome pathway. In consistence, we found that FasL-induced binding of β-catenin with the P-gp promoter depends on ERK1/2 MAPK-GSK3β signaling. Most importantly, a positive intercorrelation among the expression of FasL, β-catenin, and P-gp was noted in human GI cancer samples, further supporting the results from *in vitro* experiments. However, FasL-induced expression of Snail and Twist during EMT [7] seems not involved in the regulation of P-gp expression, as knockdown of either Snail or Twist by shRNA didn't affect P-gp expression after FasL stimulation (data not shown).

microRNAs (miRNAs) are small and endogenous noncoding RNAs that can simultaneously regulate the expression of multiple genes, primarily by binding to the 3′ untranslated region (UTR) of target mRNA and inhibiting protein translation [30]. Important roles for miRNAs including regulation of cancer proliferation and metastasis have been shown in most types of cancers [31]. miRNAs are also components of the cellular signaling circuitry that regulate the EMT program [22]. For example, miR-200 and miR-205 promote E-cadherin expression by inhibiting ZEB1 and ZEB2, and thereby help in maintaining the epithelial cell phenotype [32–34]. To date, lots of miRNAs have been proposed to target the P-gp mRNA 3′UTR and inhibit its translation, such as miR-508-5p [18], miR-451 [19], miR-145 [20], miR-298 [21], miR-1253 [21], and miR-338 [21]. We found that FasL stimulation downregulated miR-145 expression in an ERK1/2 MAPK dependent manner. We further validated that P-gp is a direct target of miR-145. Moreover, a significantly negative correlation between miR-145 and FasL or P-gp was found in human GI cancer samples. Similarly, miR-145 expression was demonstrated significantly decreased in breast, colon, stomach, and bladder cancers and also involved in the progression of these cancers[35–39]. Mitogen-activated protein kinase (MAPK) signaling is deregulated in most cancers and downregulates miR-145 expression via Ras-responsive element-binding protein (RREB1), which directly binds and suppresses miR-145 promoter [40]. However, we didn't found an induction of total or nuclear RREB1 expression following FasL treatment. Further investigation will be needed to elucidate how Fas signaling regulates miR-145 expression.

In conclusion, our study has demonstrated that, in addition to promoting cancer cell invasion and metastasis, Fas signaling enhances drug resistance by upregulating the expression of ABC transporters, especially P-gp, via increasing nuclear β-catenin through ERK1/2 MAPK-GSK3β signaling axis and downregulating miR-145. Our data provide novel insights into the molecular mechanisms behind the association between cancer metastasis and drug resistance, and will guide the strategies targeting Fas signaling for cancer treatment.

## MATERIALS AND METHODS

### Cell culture and reagents

Human colorectal cancer (CRC) SW480 and DLD1 cells, and gastric cancer (GC) AGS cells were obtained from American Type Culture Collection (Manassas, VA) and routinely maintained as previously described [7]. Serum-free medium was used in most experiments unless otherwise indicated. FasL was used at a concentration of 12.5 ng/ml as previously described [7]. U0126 (10 μM) or PD98059 (10 μM) was used to inhibit ERK1/2 MAPK signaling. Quercetin (10 μM) was used to repress the transcriptional activity of β-catenin/TCF [41]. Tariquidar (1 μM) was used to depress the P-gp activity. All inhibitors were added into the culture medium two hours before FasL treatment. Detailed information for reagents and antibodies was listed in the Supplementary Information.

### Cell transfection

Previously, two sets of shRNA constructs (*β-catenin*, *c-Jun*, or *c-Fos* shRNA) were purchased from Santa Cruz Biotechnology (Santa Cruz, CA) and GeneCopoeia (Rockville, MD), which were transduced in SW480, DLD1 and AGS cells, respectively [14, 15]. Similar results for experiments of this study were confirmed using these constructs from different companies (data not shown). *ABCB1* shRNA was also purchased from Santa Cruz Biotechnology and GeneCopoeia, and stably transfected in SW480, DLD1 and AGS cells. SW480, DLD1 and AGS cells expressing the GSK-3β S9A mutant (Addgene, Cambridge, MA), a constitutively active mutant unable to be inhibited by phosphorylation at Ser 9, was utilized as previously described [14]. The miR-145 or control precursors (GeneCopoeia) were stably transfected into SW480, DLD1 and AGS cells. Detailed information for transfectants was listed in the Supplementary Information. All procedures were conducted according to the manufacturers' protocols.

### GI cancer specimens and follow-up

GI precancerous (Colon and rectum, N=367; Stomach, N=419) and cancer samples (CRC, N=135; GC, N=143) were collected from Nanfang Hospital (Guangzhou, China). None of the patients received therapy before the study. All tissues were examined by at least two experienced pathologists and checked for the presence of tumor cells. The research protocol was approved by the Ethics Committee of Nanfang Hospital and consent was acquired from all patients for the study. Follow-up data was available for all patients with GI cancer. Correlation between clinicopathologic parameters and expression of the investigated molecules in GI cancer was shown in Supplementary Table 1, 2, 5 and 6.

### Analysis of chemotherapy response

Patients' medical records, including endoscopy and CT, were retrospectively reviewed from their electronic medical history. The chemotherapy protocols for unresectable metastatic CRC and GC patients were FOLFIRI (5-FU, leucovorin, irinotecan). Both CT and endoscopy were systematically evaluated before and after chemotherapy, principally after the fourth course. CT scans were reviewed by a radiologist with a clinician blinded to patient outcome and results whereas endoscopic images for primary tumor were evaluated by two endoscopists who were blinded to the overall effects of chemotherapy. The assessment of chemotherapy response evaluated by CT was performed according to RECIST criteria: complete response (CR), partial response (PR), stable disease (SD), and progressive disease (PD) [42]. FasL expression in human GI cancer samples was examined by both qRT-PCR and immunohistochemistry. Samples with consistent results by both methods were used (CRC, N=63; GC, N=60) while samples with inconsistent results were excluded.

### Luciferase reporter assay

The P-gp promoter reporter (GeneCopoeia) or 3′UTR miRNA target clone (GeneCopoeia), a dual-reporter system with Gaussia Luciferase (GLuc) and Secreted Alkaline Phosphatase (SEAP), was introduced into GI cancer cells using Endofectin^TM^ (GeneCopoeia). The predicted miR-145 and β-catenin binding sites on P-gp 3′UTR region and promoter were mutated using Quick Change Site-Directed Mutagenesis Kit (Agilent, Santa Clara, CA), respectively. Luciferase activity was assessed with the Secrete-Pair™ Dual Luminescence Assay Kit (GeneCopoeia) as previously described [14, 15].

### Cell survival analysis

GI cancer cells in serum-free media were seeded in 96-well plate (10000 cells/well) overnight. 24 hours after treatment with FasL, chemotherapeutic agent (5-Fu: 10 μg/ml, SN-38: 2 μM, Oxaliplatin: 20 μM) was added into the media for 48 hours. Cell survival was analyzed by the CellTiter-Blue^®^Cell Viability Assay (Promega, Madison, WI).

### Rhodamine 123 (Rh-123) efflux assay

The transport activity of P-gp was examined by testing the efflux of rhodamine 123 as described [43]. Briefly, cells were incubated with 2 μM of Rh-123 for 30 min at 37°C after FasL treatment for 3 days. At the end of incubation, cells were washed 3 times with PBS to remove free Rh-123 and kept in dye-free medium at 37°C. The fluorescence of remaining Rh-123 inside the cells was analyzed by Flow cytometry (BD FACS Calibur, Franklin Lakes, NJ).

### Chromatin immunoprecipitation (ChIP)

ChIP assay was performed with EZ-ChIP kit (Millipore, Bedford, MA) as previously described [15]. The purified DNA was used as template and amplified with the following primer set, which are specific for the first β-catenin binding site in the P-gp promoter next to transcription starting site: 5′-tgctgaagaaagaccactgca-3′ and 5′-aaacgcgcatcagctgaatc-3′.

### Quantitative real-time PCR (qRT-PCR), immunoblot, and immunohistochemistry

Immunoblot [7], immunohistochemistry [7,14] and qRT-PCR [15] were performed as previously described. qRT-PCR primers for miR-145, snRNA U6, 16 ABC transporters, GAPDH and FasL were purchased from GeneCopoeia.

### Statistical analysis

Statistical analysis was conducted using SPSS 17.0 (SPSS Inc., Chicago, IL). Difference in the expression of each molecule in ranked data was calculated using Chi-square test. Significance between changes in different groups detected by qRT-PCR, cell viability assay and luciferase reporter assay was evaluated by one-way analysis of variance (ANOVA), while least-significant difference (LSD) test was used for multiple comparisons. Correlation coefficient was calculated by the Spearman method. Survival curves were generated according to follow-up data with Kaplan-Meier method, and comparison between cumulative survival rates was performed using log-rank test. P-values less than 0.05 were considered statistically significant.

## SUPPLEMENTARY METHODS, TABLES AND FIGURES


