# Supplementary material for: Fas signaling promotes chemoresistance in gastrointestinal cancer by up-regulating P-glycoprotein
Source: Oncotarget. 2014 Oct 15;5(21):10763–77. doi: 10.18632/oncotarget.2498 (PMC4279408; doi:10.18632/oncotarget.2498)
Supplement: Supplementary file 1 [file oncotarget-05-10763-s001.pdf]

SUPPLEMENTARY METHODS, TABLES AND FIGURES

Antibodies, reagents and transfectants

Antibodies used in immunoblot assay: anti-P-glycoprotein (ab170903, Abcam, Cambridge, MA), anti-GAPDH (sc-32233, Santa Cruz Biotechnology), anti-Histone 2A.X (ab11175, Abcam), anti-MRP1 (ab180960, Abcam), anti-FasL (sc-823, Santa Cruz Biotechnology), anti-β-catenin (#8480, Cell Signaling Technology, Danvers, MA).

Antibodies used in immunohistochemistry assay: anti-FasL (sc-823, Santa Cruz Biotechology), and anti-β-catenin (#8480, Cell Signaling Technology), anti-P-glycoprotein (ab170903, Abcam).

Antibody used in ChIP assay: anti-β-catenin (#8480, Cell Signaling Technology).

Reagents: FasL (ALX-522-001-3010, Alexis, San Diego, CA), U0126 (#9903, Cell Signaling Technology), PD98095 (#9900, Cell Signaling Technology), Quercetin (Q4951, Sigma-Aldrich, St. Louis, MO), Tariquidar (S8028, Selleckchem, Houston, TX), 5-Fu (F-6627, Sigma-Aldrich), Oxaliplatin (O9512, Sigma-Aldrich), SN-38 (H0165, Sigma-Aldrich).

Transfectants: ABCB1 shRNA lentiviral particle (sc-29395-V, Santa Cruz Biotechnology), β-catenin shRNA lentiviral particle (sc-29209-V, Santa Cruz Biotechnology), c-Jun shRNA lentiviral particle (sc-29223-V, Santa Cruz Biotechnology), c-Fos shRNA lentiviral particle (sc-29221-V, Santa Cruz Biotechnology), Control shRNA Lentiviral Particles (sc-108080, Santa Cruz Biotechnology); ABCB1 shRNA clone (HSH013100, GeneCopoiea), β-catenin shRNA clone (HSH054811, GeneCopoiea), c-Jun shRNA clone (HSH009853, GeneCopoiea), c-Fos shRNA clone (HSH006188, GeneCopoiea), Scramble shRNA control clone (CSHCTR001, GeneCopoiea); miR-145 precursor (HmiR0085-MR03, GeneCopoiea), Precursor miRNA scrambled control clones (CmiR0001-MR03, GeneCopoiea); miR-145 inhibitor (HmiR-AN0191- AM03, GeneCopoiea), miRNA inhibitor control clones (CmiR-AN0001-AM03, GeneCopoiea), GSK-3β S9A mutant construct (Plasmid 14128, Addgene, Cambridge, MA), control construct (Plasmid 1764, Addgene).

TABLE S1-S4 FOR QRT-PCR ANALYSIS OF FASL, MIR-145 AND P-GP

Supplementary Table S1. Correlation of clinicopathologic parameters with FasL, miR-145 and P-gp in colorectal cancer

Clinicopathologic parameters	Number (135)	FasL		*P*	miR-145		*P*	P-gp		*P*
		-/+ (64)	++/+++ (71)		-/+ (63)	++/+++ (72)		-/+ (70)	++/+++ (65)	
**Age**				**0.765**			**0.890**			**0.909**
<60	72	35	37		34	38		37	35	
>60	63	29	34		29	34		33	30	
**Gender**				**0.670**			**0.347**			**0.929**
Male	67	33	34		34	33		35	32	
Female	68	31	37		29	39		35	33	
**Site**				**0.373**			**0.963**			**0.312**
Colon	79	40	39		37	42		38	41	
Rectum	56	24	32		26	30		22	34	
**Differentiation**				**0.655**			**0.647**			**0.404**
Well	34	18	16		17	17		18	16	
Moderate	57	31	26		28	29		26	31	
Poor	44	20	24		18	26		26	18	
**Dukes' stage**				**<0.01**			**<0.01**			**<0.01**
A	60	48	12		15	45		43	17	
D	75	16	59		48	27		27	48	

Significance was noted between Dukes' stage A and D for expression of FasL, miR-145, and P-gp.

Supplementary Table S2. Correlation of clinicopathologic parameters with FasL, miR-145 and P-gp in gastric cancer

Clinicopathologic parameters	Number (143)	FasL		*P*	miR-145		*P*	P-gp		*P*
		-/+ (67)	++/+++ (76)		-/+ (70)	++/+++ (73)		-/+ (69)	++/+++ (74)	
**Age**				**0.603**			**0.465**			**0.648**
<60	65	32	33		34	31		30	35	
>60	78	35	43		36	42		39	39	
**Gender**				**0.532**			**0.924**			**0.950**
Male	75	37	38		37	38		36	39	
Female	68	30	38		33	35		33	35	
**Site**				**0.886**			**0.300**			**0.353**
Proximal	42	20	22		23	19		24	18	
Middle	49	24	25		26	23		23	26	
Distal	52	23	29		21	31		22	30	
**Differentiation**				**0.917**			**0.948**			**0.544**
Well	40	18	22		20	20		21	19	
Moderate	53	26	27		25	28		27	26	
Poor	50	23	27		25	25		21	29	
**p-Stage**				**<0.01**			**<0.01**			**<0.01**
I	75	54	21		15	60		**59**	16	
II-IV	68	13	55		55	13			10	**58**

Significance was noted between p-stage I and II-IV for expression of FasL, miR-145, and P-gp.

Supplementary Table S3. Correlation between FasL, miR-145 and P-gp expression in colorectal cancer

	N=135	miR-145 expression -(28)	+(35)	++(34)	+++(38)	Correlation coefficient
FasL expression	-(29)	4	3	10	12	R =-0.342 (*P*<0.01)
	+(35)	2	5	16	12	
	++(33)	10	12	6	5	
	+++(38)	12	15	2	9	

	N=135	P-gp expression -(34)	+(36)	++(32)	+++(33)	Correlation coefficient
FasL expression	-(29)	12	11	4	2	R =0.380 (*P*<0.01)
	+(35)	11	16	5	3	
	++(33)	3	4	11	15	
	+++(38)	8	5	12	13	

	N=135	P-gp expression -(34)	+(36)	++(32)	+++(33)	Correlation coefficient
miR-145	-(28)	5	3	9	11	R =-0.379 (*P*<0.01)
	+(35)	4	6	11	14	
	++(34)	12	12	4	6	
	+++(38)	13	15	8	2	

Supplementary Table S4. Correlation between FasL, miR-145 and P-gp expression in gasric cancer

	N=143	miR-145 expression -(32)	+(38)	++(38)	+++(35)	Correlation coefficient
FasL expression	-(16)	3	2	7	4	R =-0.322 (*P*<0.01)
	+(51)	6	8	17	20	
	++(49)	14	19	7	9	
	+++(27)	9	9	7	2	

	N=143	P-gp expression -(30)	+(39)	++(36)	+++(38)	Correlation coefficient
FasL expression	-(16)	5	7	2	2	R =0.394 (*P*<0.01)
	+(51)	14	24	6	7	
	++(49)	7	6	20	16	
	+++(27)	4	2	8	13	

	N=143	P-gp expression -(30)	+(39)	++(36)	+++(38)	Correlation coefficient
miR-145	-(32)	4	6	12	10	R =-0.246 (*P*<0.01)
	+(38)	5	8	13	12	
	++(38)	10	13	6	9	
	+++(35)	11	12	5	7	

TABLE S5-S8 FOR IMMUNOHISTOCHEMISTRY ANALYSIS OF FASL, P-GP AND B-CATENIN

Supplementary Table S5. Correlation of clinicopathologic parameters with FasL, P-gp and β-catenin in colorectal cancer

Clinicopathologic parameters	Number (135)	FasL		*P*	P-gp		*P*	Nuclear β-catenin		*P*
		-/+ (68)	++/+++ (67)		-/+ (66)	++/+++ (69)		-/+ (72)	++/+++ (63)	
**Age**				**0.800**			**0.680**			**0.628**
<60	72	37	35		34	38		37	35	
>60	63	31	32		32	31		35	28	
**Gender**				**0.547**			**0.266**			**0.800**
Male	67	32	35		36	31		35	32	
Female	68	36	32		30	38		37	31	
**Site**				**0.441**			**0.828**			**0.963**
Colon	79	42	37		38	41		42	37	
Rectum	56	26	30		28	28		30	26	
**Differentiation**				**0.522**			**0.784**			**0.942**
Well	34	20	14		18	16		19	15	
Moderate	57	27	30	**26**		31		30	27	
Poor	44	21	23		22	22		23	21	
**Dukes' stage**				**<0.01**			**<0.01**			**<0.01**
A	60	44	16		43	17		46	14	
D	75	24	51		23	52		26	49	

Significance was noted between Dukes' stage A and D for expression of FasL, P-gp, and β-catenin.

Supplementary Table S6. Correlation of clinicopathologic parameters with FasL, P-gp and β-catenin in gastric cancer

Clinicopathologic parameters	Number (143)	FasL		*P*	P-gp		*P*	Nuclear β-catenin		*P*
		-/+ (73)	++/+++ (70)		-/+ (68)	++/+++ (75)		-/+ (73)	++/+++ (70)	
**Age**				**0.541**			**0.715**			**0.285**
<60	65	35	30		32	33		30	35	
>60	78	38	40		36	42		43	35	
**Gender**				**0.566**			**0.911**			**0.923**
Male	75	40	35		36	39		38	37	
Female	68	33	35		32	36		35	33	
**Site**				**0.864**			**0.935**			**0.983**
Proximal	42	20	22		19	23		21	21	
Middle	49	26	23		24	25		25	24	
Distal	52	27	25		25	27		27	25	
**Differentiation**				**0.866**			**0.179**			**0.037**
Well	40	21	19		23	17		26	14	
Moderate	53	28	25		26	27		28	25	
Poor	50	24	26		19	31		19	31	
**p-Stage**				**<0.01**			**<0.01**			**<0.01**
I	75	50	25		54	21		57	18	
II-IV	68	23	45		14	54		16	52	

Significance was noted between p-stage I and II-IV for expression of FasL, P-gp, and β-catenin. Nuclear expression of β-catenin was also noticeably distributed in cancer differentiation.

Supplementary Table S7. Correlation between FasL, P-gp and β-catenin expression in colorectal cancer

	N=135	FasL expression -(30)	FasL expression +(38)	FasL expression ++(33)	FasL expression +++(34)	Correlation coefficient
P-gp expression	-(32)	12	13	4	3	R =0.441 (P<0.01)
	+(34)	10	16	5	3	
	++(36)	4	5	12	15	
	+++(33)	4	4	12	13	

	N=135	FasL expression -(30)	FasL expression +(38)	FasL expression ++(33)	FasL expression +++(34)	Correlation coefficient
β-catenin expression	-(27)	10	13	3	1	R =0.513 (P<0.01)
	+(45)	15	19	5	6	
	++(39)	2	3	18	16	
	+++(24)	3	3	7	11	

	N=135	β-catenin expression -(27)	β-catenin expression +(45)	β-catenin expression ++(39)	β-catenin expression +++(24)	Correlation coefficient
P-gp expression	-(32)	13	12	3	4	R =0.454 (P<0.01)
	+(34)	10	20	2	2	
	++(36)	2	6	16	12	
	+++(33)	2	7	18	6	

CRC: Colorectal cancer

Supplementary Table S8. Correlation between FasL, P-gp and β-catenin expression in gastric cancer

	N=143	FasL expression -(15)	FasL expression +(58)	FasL expression ++(28)	FasL expression +++(42)	Correlation coefficient
P-gp expression	-(33)	6	20	2	5	R =0.519 (*P*<0.01)
	+(35)	7	25	1	2	
	++(39)	1	8	12	18	
	+++(36)	1	5	13	17	

	N=143	FasL expression -(15)	FasL expression +(58)	FasL expression ++(28)	FasL expression +++(42)	Correlation coefficient
β-catenin expression	-(37)	7	21	3	6	R =0.442 (*P*<0.01)
	+(36)	3	28	1	4	
	++(44)	2	6	17	19	
	+++(26)	3	3	7	13	

	N=143	β-catenin expression -(37)	β-catenin expression +(36)	β-catenin expression ++(44)	β-catenin expression +++(26)	Correlation coefficient
P-gp expression	-(33)	12	15	4	2	R =0.486 (*P*<0.01)
	+(35)	15	13	5	2	
	++(39)	6	5	20	8	
	+++(36)	4	3	15	14	

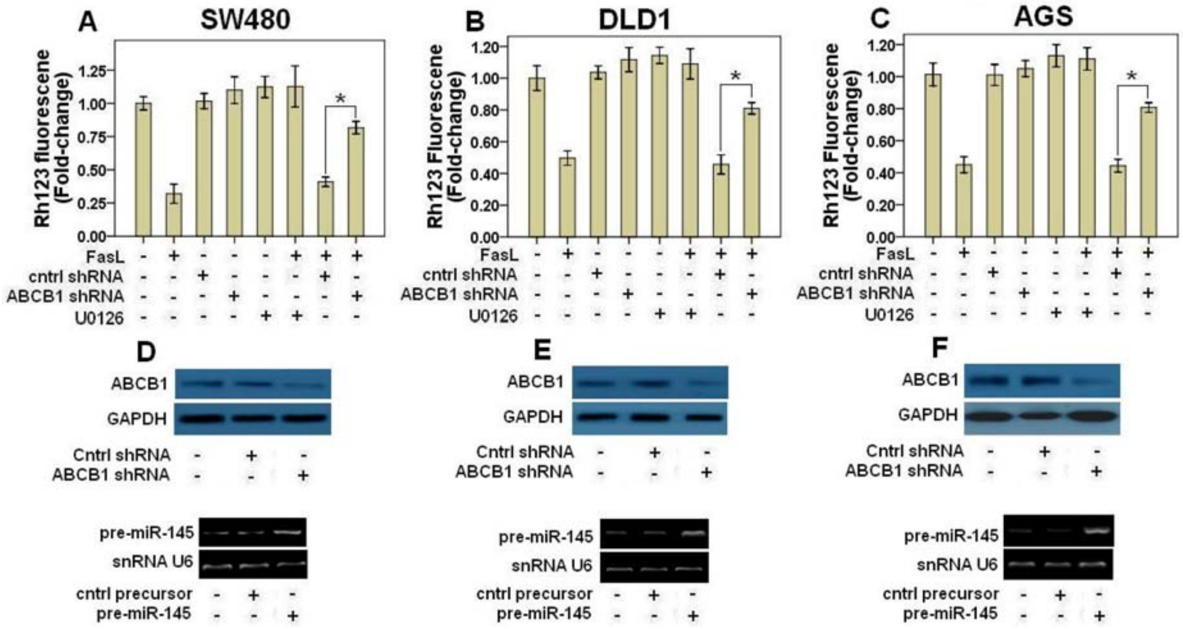

Supplementary Figure S1: SW480 (A), DLD1 (B), and AGS (C) expressing ABCB1 shRNA or control shRNA were treated with or without FasL for 24 hours, and then 2 µM of Rh-123 was added into the media. The fluorescence of remaining Rh-123 in the cells was analyzed by flow cytometry. U0126 was added 2 hours before FasL stimulation. The efficacy of gene tranfection in SW480 **(D)**, DLD1 **(E)**, and AGS **(F)** was shown. **(A-C)** Data are represented as fold-change ± SD compared to control cells. Experiments were performed in triplicate. $^{*}P < 0.05$.

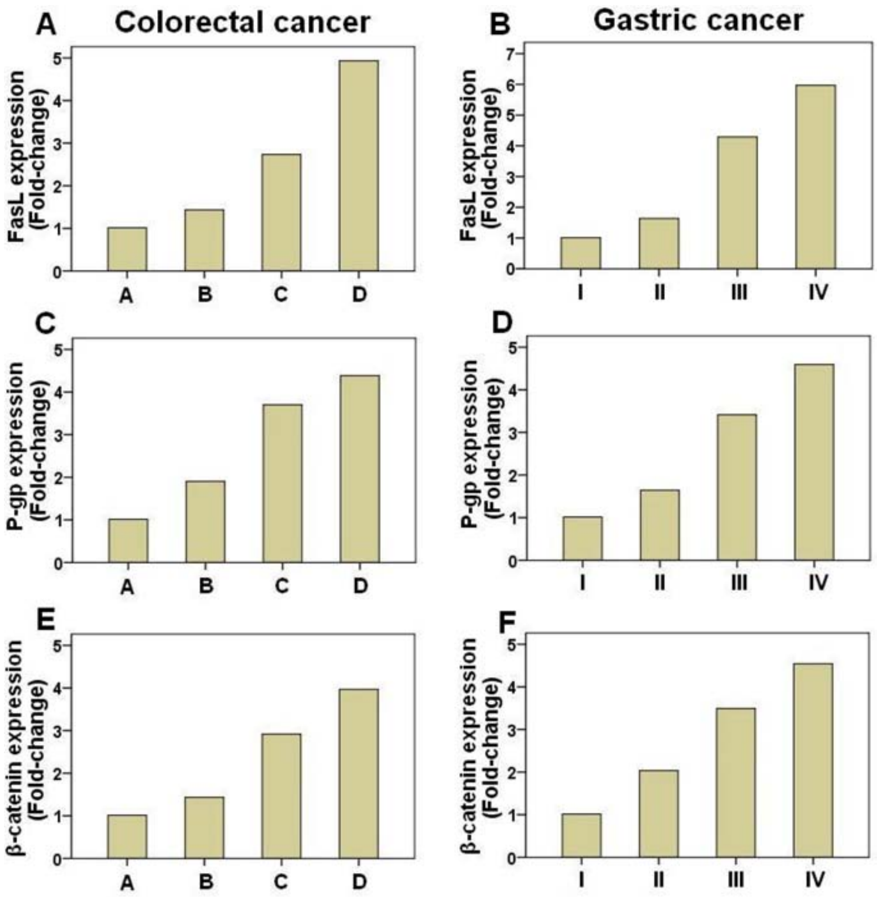

Supplementary Figure S2: Expression of FasL, P-gp, and β-catenin in GI cancer specimens was detected by immunoblot (N=20). Densitometric analysis was performed using Quantity One software. GAPDH was used as an internal control, and the expression of each molecule was normalized to GAPDH expression for each sample. All data are represented as fold-change compared to control group.

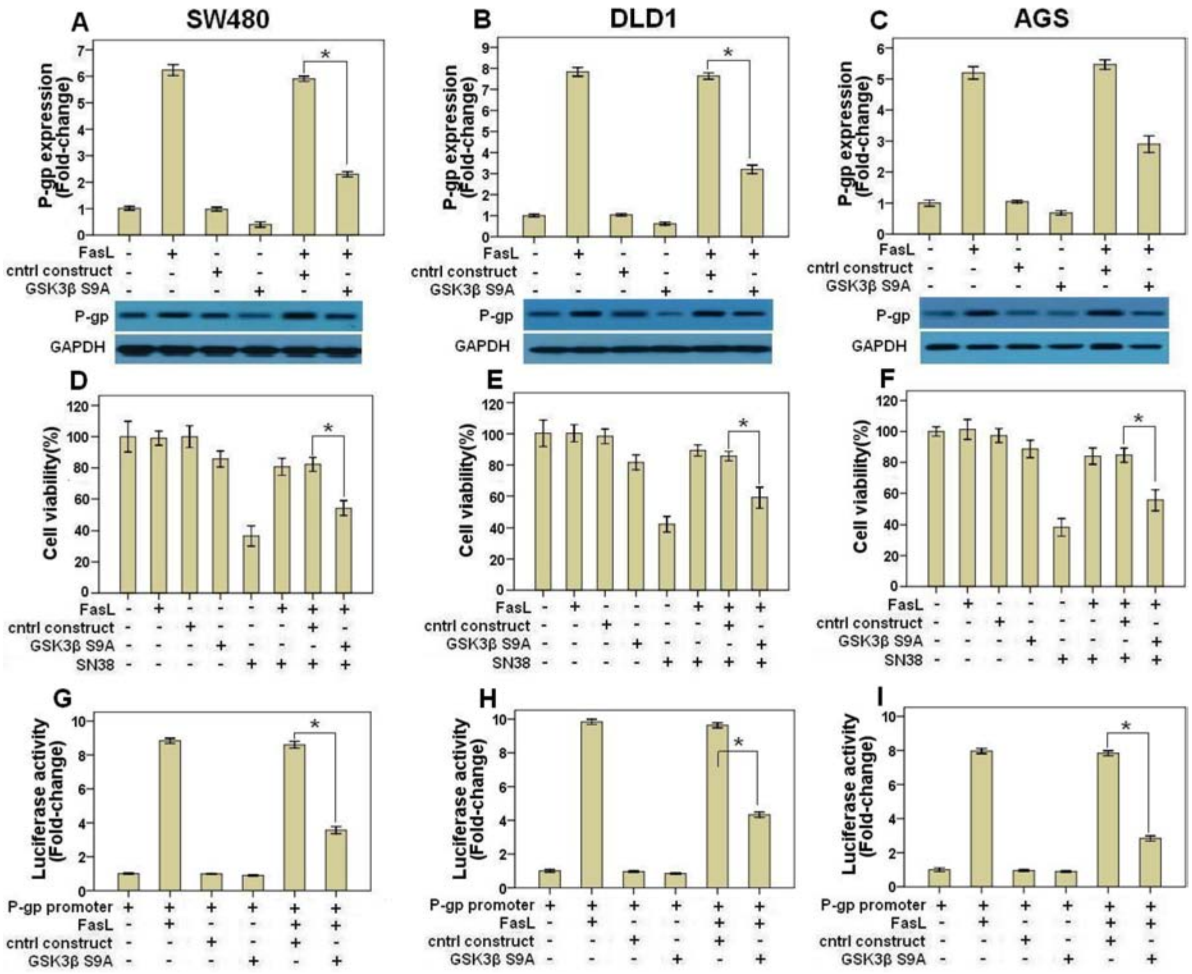

Supplementary Figure S3: FasL-induced inhibition of GSK3β promotes P-gp expression. SW480 **(A),** DLD1 **(B),** and AGS **(C)** cells stably expressing GSK3β S9A or control construct were treated with FasL for 24 or 72 hours, and then qRT-PCR or immunoblot was conducted. SW480 **(D),** DLD1 **(E),** and AGS **(F)** cells stably expressing GSK3β S9A or control construct were seeded in 96-well plate (10000 cells/well) and cultured overnight, and then treated with FasL for 24 hours. After that, cells were treated with SN-38 for 48 hours, and then cell viability was analyzed. P-gp promoter reporter was transduced into SW480 **(G),** DLD1 **(H),** and AGS **(I)** cells stably expressing either GSK3β S9A or control construct, and luciferase activity was assessed after FasL treatment for 12 hours. All data are represented as fold-change ± SD compared to control cells. Experiments were performed in triplicate. *$P < 0.05$.

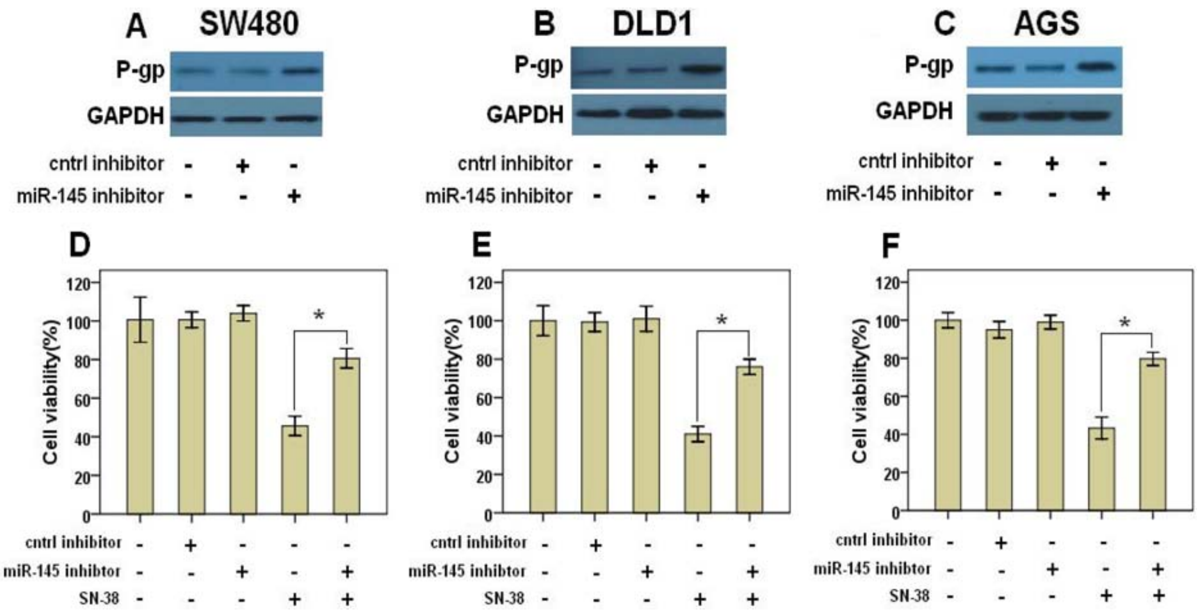

Supplementary Figure S4: Downregulation of miR-145 promotes P-gp expression and inhibits cell apoptosis induced by SN-38. miR-145 inhibitor or control inhibitor was stably transduced into SW480 **(A),** DLD1 **(B),** and AGS **(C).** P-gp was tested by immunoblot. SW480 **(D),** DLD1 (E), and AGS (F) cells, expressing miR-145 inhibitor, were seeded in 96-well plate (10000 cells/well) and cultured overnight. Then, cells were treated with SN-38 (2 μM) for additional 48 hours, and cell viability was analyzed by CellTiter-Blue®Cell Viability Assay. (D-F) Data are represented as fold-change ± SD compared to control cells. Experiments were performed in triplicate. *P < 0.05.

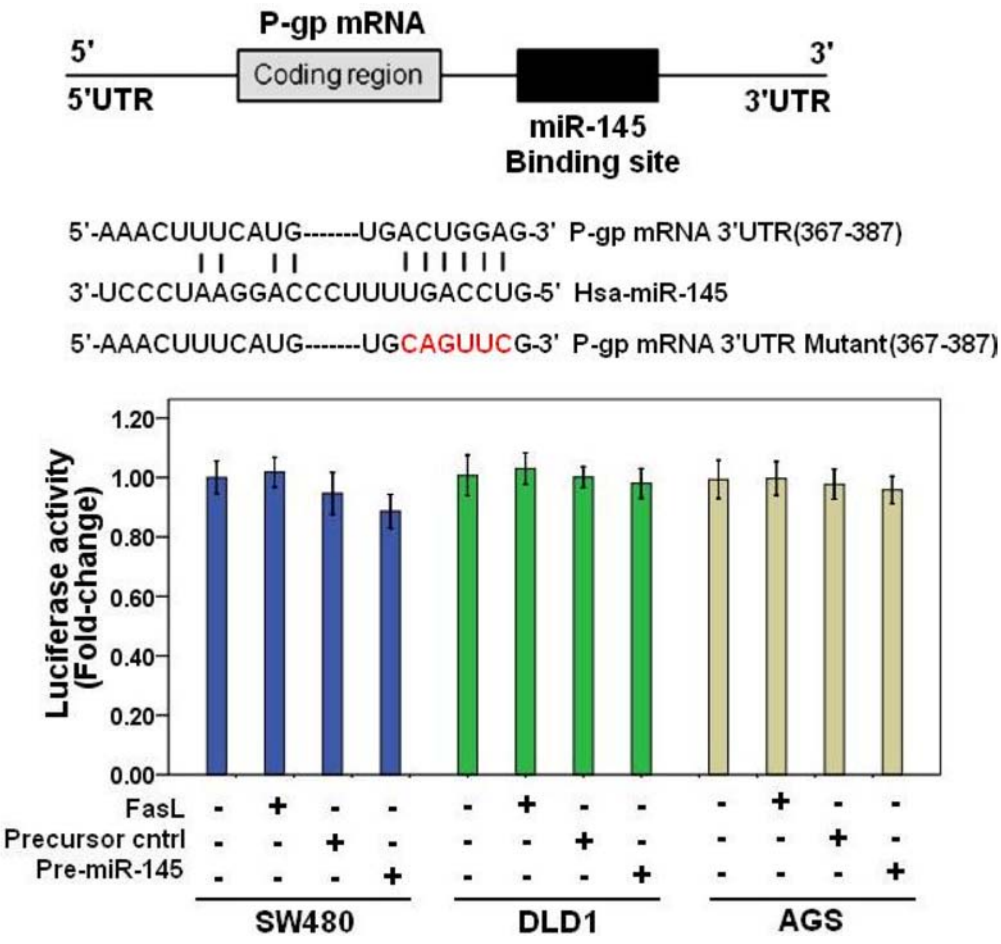

Supplementary Figure S5: miR-145 targets the predicted binding site of p-GP 3′UTR region (www.microrna.org, www.targetscan.org). Figures 6D-6F showed that the miR-145 precursor significantly inhibited P-gp 3′UTR reporter activity and protein expression. Then a mutant construct in which miR-145 binding site of P-gp 3′UTR region was mutated was generated using Quick Change Site-Directed Mutagenesis Kit (Agilent). This construct was transduced into GI cancer cells expressing miR-145 precursor or control precursor, and then luciferase activity was assessed after FasL treatment for 12 hours. All data are represented as fold-change ± SD compared to control cells. Experiments were performed in triplicate.
